# PreserFlo MicroShunt® exposure: a case series

**DOI:** 10.1186/s12886-021-02032-z

**Published:** 2021-07-10

**Authors:** Roxane Bunod, Mathieu Robin, Juliette Buffault, Chafik Keilani, Antoine Labbé, Christophe Baudouin

**Affiliations:** 1 Department of Ophthalmology III, Quinze-Vingts National Ophthalmology Hospital, 28 rue de Charenton, 75012 Paris, France; 2 Quinze-Vingts National Ophthalmology Hospital, IHU FOReSIGHT, INSERM-DGOS CIC 1423, 17 rue Moreau, F-75012 Paris, France; 3 Sorbonne Universités, INSERM, CNRS, Institut de la Vision, 17 rue Moreau, 75012 Paris, France; 4 Department of Ophthalmology, Ambroise Paré Hospital, AP-HP, 9 avenue Charles de Gaulle, 92100 Boulogne-Billancourt, France

**Keywords:** Microshunt, InnFocus, PreserFlo, MIGS

## Abstract

**Background:**

PreserFlo® MicroShunt (PM) (also known as InnFocus® MicroShunt) is a subconjunctival stent implanted *ab externo* via a minimally invasive surgical procedure. The current indication is progressive, mild to moderate, open angle glaucoma uncontrolled on topical medications. According to the literature, adverse events are rare, mild and transient.

**Case presentation:**

Two cases of stand-alone PreserFlo MicroShunt® implantation in patients with uncontrolled open-angle glaucoma are reported. Exposure occurred 7 days and 3 months respectively after implantation. These cases shared common features including preexisting blepharitis and the lack of a Tenon’s flap. In both cases, removal of the device was required after several attempts at repair.

**Conclusions:**

PreserFlo MicroShunt® exposure is a potentially vision-threatening complication because of the risk of endophthalmitis. Potential risk factors include the absence of a Tenon’s flap and pre-existing ocular surface inflammation. Ocular surface inflammation should be detected and treated prior to PM implantation. If a deficiency in Tenon’s capsule is noted intraoperatively, close monitoring should be performed because of the higher risk of PM exposure.

## Background

Over the past decade, minimally invasive glaucoma surgery (MIGS) has emerged and currently includes various devices such as Schlemm’s canal stents (iStent®, Glaukos Corporation, San Clemente, CA, USA; Hydrus® Microstent, Ivantis, Inc., Irvine, CA, USA), suprachoroidal stent (iStent Supra®, Glaukos Corporation, San Clemente, CA, USA), and subconjunctival stents (XEN45® Gel Stent, Allergan Inc., Irvine, CA, USA; PreserFlo® MicroShunt, Santen, Osaka, Japan) [1]. The mechanism of intraocular pressure (IOP) reduction by subconjunctival stents is similar to the gold standard glaucoma surgery, trabeculectomy, which is creation of a new pathway to drain aqueous humor from the anterior chamber to a subconjunctival space known as a “filtering bleb.” The PreserFlo® MicroShunt (PM) (also known as InnFocus® MicroShunt) is an 8.5 mm-long stent (outer diameter 350 μm; internal lumen diameter 70 μm) made of polystyrene-block-isobutylene-block- styrene (SIBS) and implanted *ab externo* after creating a conjunctival flap and a small scleral tunnel [2]. The procedure can be performed as a standalone procedure or in combination with cataract surgery. The current indication is progressive, mild to moderate, open angle glaucoma uncontrolled on topical medications. According to the literature, adverse events are rare, the most frequent being transient hypotony, flat anterior chamber, choroidal detachment, and hyphema [3–7]. All of these complications are transient and resolve spontaneously without vision threatening events. In the present study, we report two cases of PM extrusion and review the literature concerning the risk factors and management of stent exposure following subconjunctival MIGS surgeries.

## Case description 1

A 53-year-old man with a history of bilateral open-angle glaucoma treated for 7 years with IOP-lowering eyedrops (Timolol maleate 0.5% + Travoprost 0.004% fixed combination, Duotrav®, Novartis, Basel, Switzerland) presented with uncontrolled IOP and progressive glaucoma. Best corrected visual acuity was 20/20 in both eyes, and the IOP was 30 mmHg in the right eye and 24 mmHg in the left eye (central corneal thickness (CCT) of 500 *μ* m and 511 *μ* m respectively). He also had a history of bilateral blepharitis and moderate dry eye treated for several years with artificial tears and azithromycin 15 mg/g ophthalmic solution (Azyter®, Thea Pharmaceuticals, Clermont-Ferrand, France). A stand-alone PM implantation was scheduled for the right eye. The procedure was performed under sub-Tenon’s anesthesia. After a conjunctival peritomy in the superior quadrant, the conjunctiva was dissected carefully from the underlying episclera, and the absence of Tenon’s layer was noted. After gentle cautery, 0.2 mg/ml mitomycin C (MMC) was applied using soaked sponges under the conjunctival flap and washed out after 2 min. A scleral pocket was created 3 mm posterior to the limbus, and a 28 G needle was passed through the scleral pocket into the anterior chamber. The PM was inserted through the tunnel and positioned correctly. Aqueous humor flow was visualized through the distal end of the device. Because of the absence of Tenon’s capsule, the conjunctiva exclusively was pulled over the PM and sutured back to the limbus using 8–0 Polysorb™ (Covidien, Dublin, Ireland) sutures made of Lactomer™ (glycolide/lactide copolymer coated with calcium stearoyl lactylate). At the conclusion of the surgical procedure, the PM was properly positioned with a filtering bleb and no leakage.

**Fig. 1:**
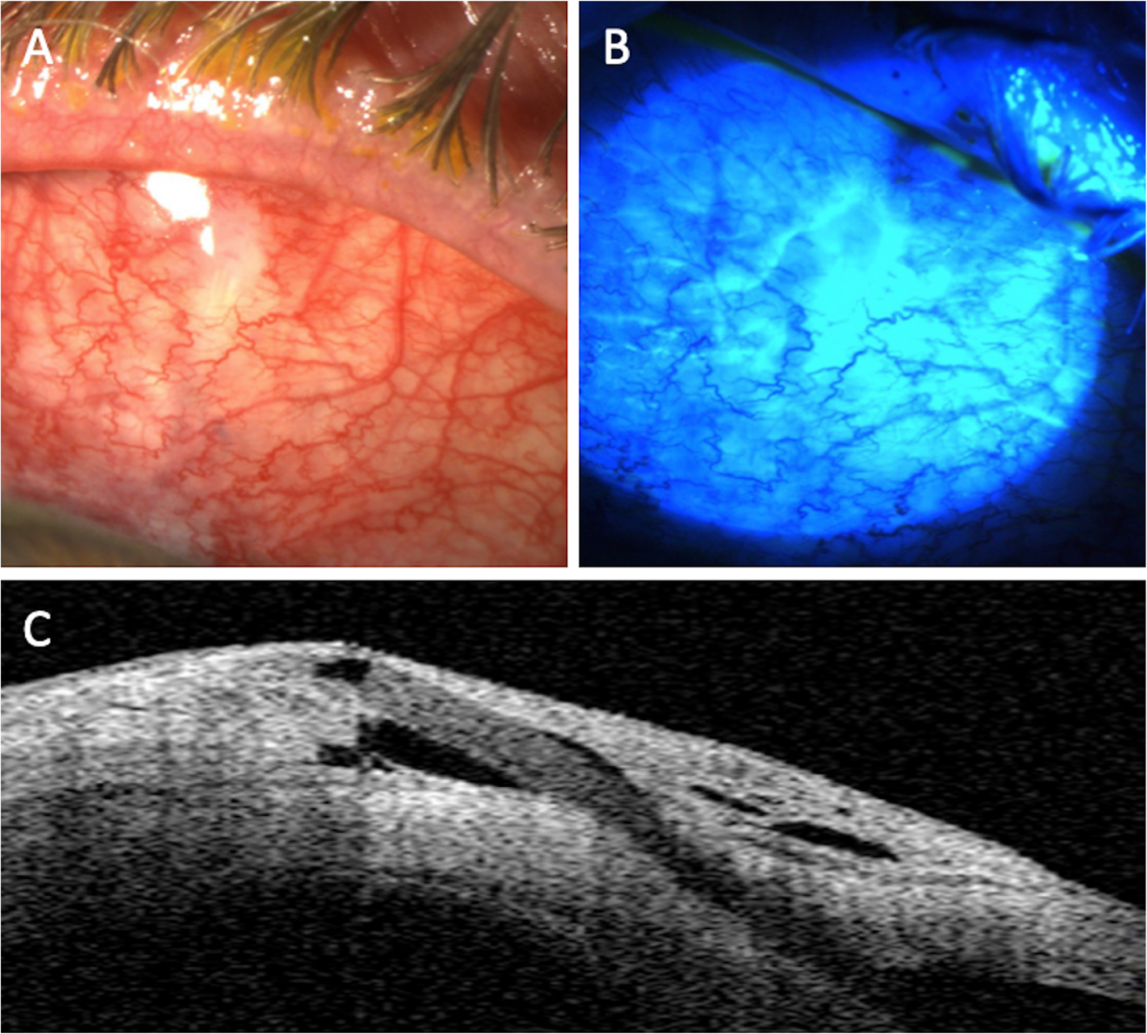
**A** Slit lamp photograph showing the exposed distal tip of the PreserFlo MicroShunt®. Note the presence of severe blepharitis associated with conjunctival inflammation. **B** Seidel sign is present after fluorescein instillation. **C** Anterior segment-OCT showing the superficial position of the PreserFlo MicroShunt®

**Fig. 2:**
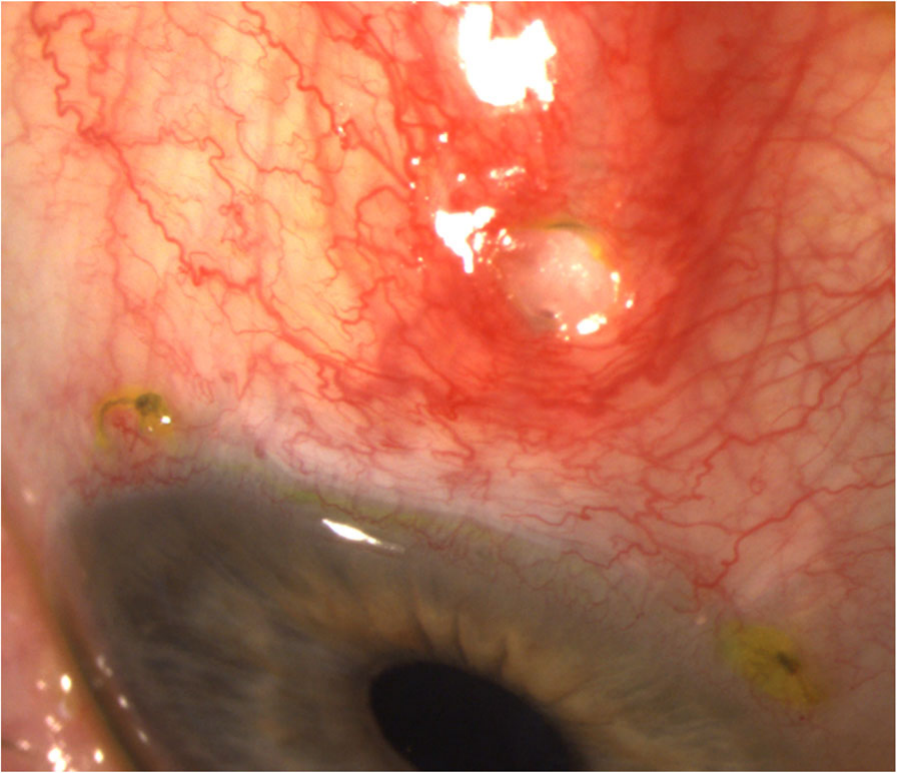
Slit lamp photograph taken 10 days after the second surgical revision showing conjunctival sutures and recurrence of the PreserFlo MicroShunt® exposure

The 1 day postoperative examination revealed an IOP of 10 mmHg in the right eye. Slit lamp examination showed the PM device protruding correctly into the anterior chamber with an elevated filtering bleb. It was noted that the outer tip of the tube was inclined slightly anteriorly under the conjunctiva, but without extrusion. Postoperative treatment consisted of dexamethasone 0.1% + tobramycin 0.3% (Tobradex®, Novartis) eyedrops and vitamin A ointment at night. Seven days after surgery, the IOP was 8 mmHg, and slit lamp examination revealed an erosion of the conjunctiva overlying the distal end of the tube. Fluorescein staining confirmed extrusion of the PM with a spontaneous positive Seidel sign (Fig. 1). A surgical revision was scheduled the same day under sub-Tenon’s anesthesia. After limbal conjunctival dissection, the PM tube was exposed and fixed to sclera with a 10–0 nylon suture buried into the sclera so that the distal end of the tube followed the scleral curvature. The conjunctiva was sutured back to the limbus with 8–0 Polysorb™ (Covidien) braided absorbable sutures, and the conjunctival defect overlying the distal end of the PM was sutured using 10–0 Vicryl™ (Ethicon, Somerville, NJ, USA) absorbable sutures. A second revision was performed 15 days later because of recurrence of the Seidel sign at the site of the sutured conjunctival defect, and this defect was resutured with 10–0 Vicryl™. Ten days later, half of the PM was protruding through the conjunctiva (Fig. 2). Urgent removal of the device was performed along with conjunctival suturing. The IOP increased to 34 mmHg the day after the surgery, and the patient received oral acetazolamide and IOP lowering drops. A temporal trabeculectomy was performed 1 month later after complete conjunctival healing. Three months after the trabeculectomy, the IOP in the right eye was 12 mmHg with an elevated filtering bleb.

## Case description 2

**Fig. 3:**
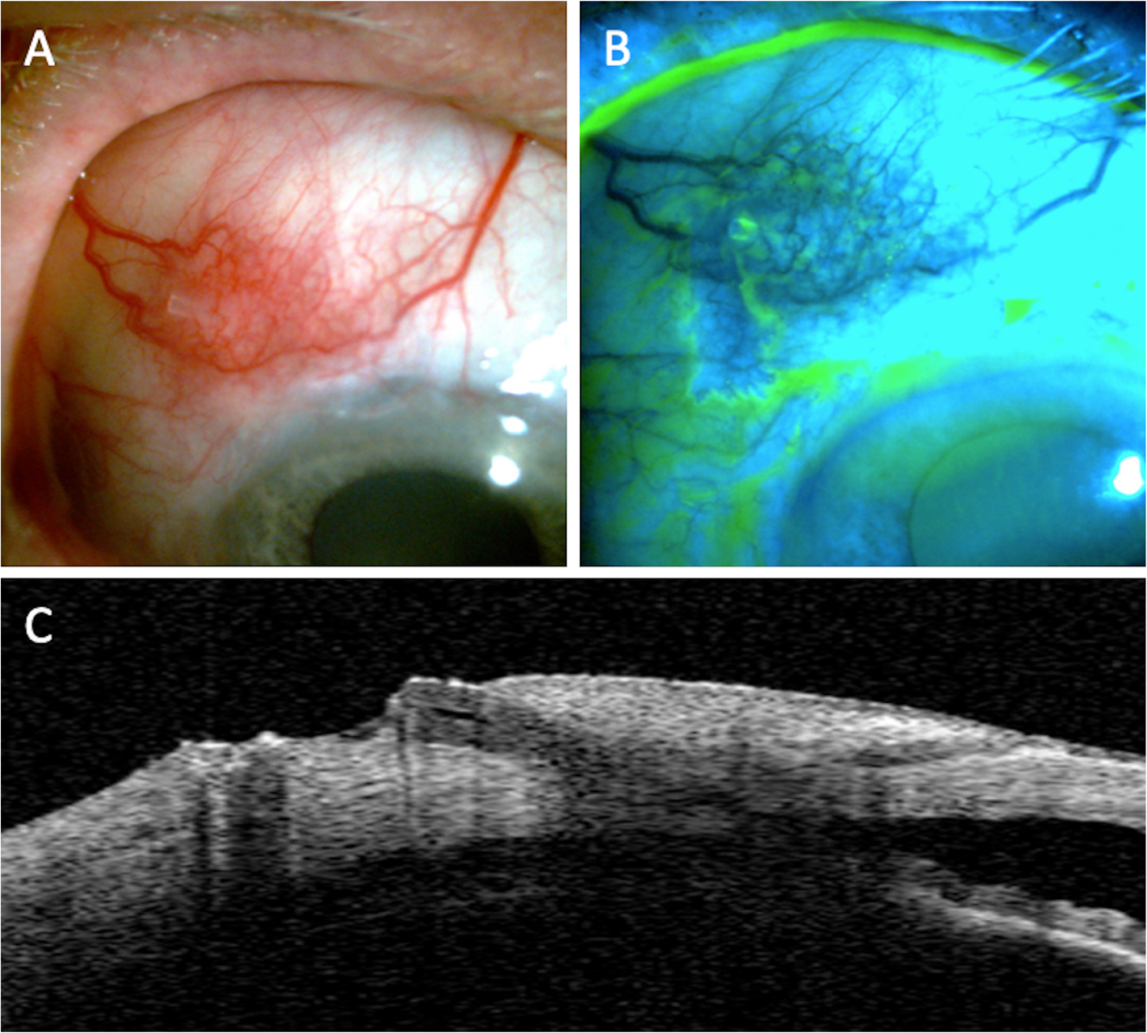
**A** Slit lamp photograph showing extrusion of the PreserFlo MicroShunt®. Note the presence of posterior blepharitis. **B** Seidel sign is present after fluorescein instillation. **C** Anterior segment-OCT showing complete erosion of the overlying conjunctiva and extrusion of the distal tip of the PreserFlo MicroShunt®

A 76-year-old man was referred to our clinic for a progressive open-angle glaucoma with uncontrolled IOP despite maximal IOP lowering medication (preservative-free dorzolamide + timolol maleate 0.5% fixed combination (Cosidime®, Santen), preservative-free latanoprost 0.005% (Monoprost®, Thea Pharma, Clermont-Ferrant, France). He had been previously treated with several topical IOP lowering medications for 20 years, including benzalkonium chloride preserved brimonidine tartrate 2% (Alphagan®, Allergan) and benzalkonium chloride preserved brinzolamide 10 mg/ml + brimonidine tartrate 2 mg/ml fixed combination (Simbrinza®, Novartis). Trabeculectomy had been performed in the right eye 8 years prior, and non-penetrating deep sclerectomy (NPDS) in both eyes 5 years prior. Visual acuity was 20/20 in both eyes, with an IOP of 45 mmHg in both eyes (CCT, 550 *μ* m and 545 *μ* m). He had severe blepharitis and dry eye associated with rosacea, treated with oral doxycycline, azithromycin ophthalmic solution and topical cyclosporine for several years. A stand-alone PM implantation was scheduled for the right eye. The procedure was performed under sub-Tenon’s anesthesia according to the previously described surgical protocol. After a limbal conjunctival incision in the superior quadrant, Tenon’s dissection revealed a very thin and retracted Tenon’s capsule. 0.2 mg/ml mitomycin C (MMC) was applied under the flap using soaked sponges and washed out after 2 min. A scleral pocket was performed 3 mm posterior to the limbus, and a tunnel connecting the scleral pocket and the anterior chamber was created with a 28 G needle. The PM was then inserted through the tunnel into the anterior chamber with the fins of the device fitting into the scleral pocket. After observation to confirm correct aqueous humor flow, Tenon’s capsule was pulled over the device but was too retracted to allow complete coverage of the PM. The conjunctival flap was sutured to the limbus using 8–0 PolysorbTM. There was no leakage at the conclusion of the procedure, and a filtering bleb was observed. Dexamethasone 0.1% + tobramycin 0.3% eyedrops (Tobradex®), indomethacin 0.1% eyedrops (Indocollyre®) and artificial tears were prescribed postoperatively. The day after the surgery, the IOP was 5 mmHg in the right eye with an elevated filtering bleb. After 10 days, the PM was properly positioned with an IOP of 10 mmHg and a large filtering bleb. The patient missed two scheduled postoperative visits, and he presented in the emergency department 3 months after the PM implantation complaining of blurred vision. The IOP was 6 mmHg in the right eye, and slit lamp examination revealed extrusion of the PM tube with a positive Seidel sign (Fig. 3). The anterior chamber was shallow with a peripheral choroidal detachment and choroidal folds. A surgical revision with amniotic membrane graft was scheduled the same day under sub-Tenon’s anesthesia. Two layers of amniotic membrane were successively applied at the tube extrusion site and fixed to the adjacent conjunctiva with 8–0 Polysorb™ (Covidien) braided absorbable suture. After 1 day, the IOP was 6 mmHg, and the amniotic membrane was covering the extrusion site with no leak. One week later, the IOP was 4 mmHg with a recurrence of PM exposure with a negative Seidel sign. A new revision was scheduled. The distal end of the PM was shortened and repositioned under the conjunctiva. The conjunctival defect was then sutured with 8–0 Polysorb™ (Covidien) braided absorbable suture. Two weeks later, the IOP was 4 mmHg in the right eye. Slit lamp examination revealed recurrence of PM extrusion associated with a positive Seidel sign. Urgent explantation of the PM was scheduled along with Xen® Gel stent (XS) implantation. One month after the surgery, the IOP was 11 mmHg in the left eye. The XS was properly positioned with a functional filtering bleb and no conjunctival erosion.

## Discussion

To reduce the risk of subconjunctival device exposure, MIGS design has evolved by reducing the outer diameter size of the devices, using more flexible material and adapting the surgical protocol. Collagen-derived gelatin XS material cross-linked with glutaraldehyde becomes flexible when hydrated – over 100 times more flexible than the silicone tube used in tube shunts [8]. Ab-interno XS implantation offers the advantage of avoiding conjunctival incision or manipulation. On the other hand, PM implantation requires a conjunctival incision and creation of a scleral pocket. However, in comparison to traditional filtering surgery, the surgical procedure is minimally invasive. The PM material “SIBS” is a recently developed thermoplastic biomaterial resisting biodegradation [9]. Several iterations of micro-shunt design were required to achieve the final design of the PM. Because the first generations of micro-shunt showed a high rate of erosion, the surgical protocol was modified with the creation of a scleral pocket to prevent the fins from eroding the conjunctiva [9]. XS exposure is estimated to occur in 2.0–4.3% of eyes in a recent literature review, and thirty-six cases of XS extrusion have been reported in the literature [10–34]. Various risks factors have been suggested by the authors. Most of the cases exhibited conjunctival fragility related to previous glaucoma surgery [23, 24], needling procedures or stent revisions [18, 27, 32–34] or even conjunctival perforation during stent implantation [16]. Lanzhofer et al. analyzed the outer stent position using optical coherence tomography (OCT) and showed that the final position of the XS can be either intra-Tenon’s, sub-Tenon’s or subconjunctival [34]. In their study, the only case of XS exposure was observed in the subconjunctival XS group. In addition, a superficial positioning of the stent was noted prior to XS exposure in four cases, highlighting the potential role of Tenon’s capsule in preventing XS exposure [25, 29, 31].

Management of XS exposure varied among the reported cases. In most of the cases, conjunctival suturing was performed, often in combination with stent repositioning or shortening [14, 17, 24, 27, 29, 30, 32, 33]. Recurrence of stent exposure after conjunctival suturing was observed in some cases, requiring additional surgical interventions [17, 29, 32, 33]. Two cases of autologous conjunctival graft along with amniotic membrane graft have been described [23, 33]. Tube removal was performed in 5 cases of XS exposure [25, 26, 28, 31, 34] and in cases of stent exposure complicated by endophthalmitis [13, 15, 16]. Transconjunctival repositioning at the slit lamp, vitamin A ointment, and a soft contact lens were used unsuccessfully in the case reported by Salinas et al. [26]. However, we have found no codified management of XS exposure in the literature.

Few cases of PM exposure have been reported in the literature. Stangos et al. reported two cases of PM exposure in 50 eyes of 40 consecutive patients with PM [35]. Durr et al. reported one patient who developed an exposed PM 6 months after the surgery in a cohort of 85 eyes [3]. However, there is a lack of information concerning the potential risk factors and surgical management of PM exposure. We describe and analyze herein the clinical presentation and management of two cases of exposed PM. These cases share common features. First, both patients presented with severe blepharitis associated with ocular surface inflammation prior to surgery. Associated eye rubbing should be ruled out, since an association between subconjunctival stent displacement and eye rubbing has been reported [36, 37]. However, none of the patients reported eye rubbing behavior. Longstanding use of IOP lowering drops, especially if they contain benzalkonium chloride, can also represent a risk factor for chronic ocular surface inflammation resulting in alteration of the conjunctival tissue [38–40]. Indeed, prior ocular inflammation has been identified as a risk factor for tube exposure after glaucoma drainage implant surgery [41]. Second, both cases were characterized by a deficiency in Tenon’s capsule. Our second patient had a history of prior NPDS, which may have contributed to changes in the conjunctiva and Tenon’s capsule. The role of previous ocular surgery in aqueous drainage device erosion has been suggested several times in the literature [42, 43]. Because of the impossibility of pulling the Tenon’s capsule over the PM tube, a simple conjunctival flap was used to cover the PM instead of a Tenon’s flap as recommended [9]. However, because of the limited number of cases, further studies are required to confirm and identify risk factors for exposure.

Management of both patients consisted of surgical repair as first-line treatment. Amniotic membrane graft, tube repositioning and conjunctival suturing were performed. However, early recurrences of PM exposure were observed in both cases. Because of the impossibility of rebuilding a Tenon’s flap and the high risk of bleb fibrosis after repair techniques, the PM was removed in both patients. The procedure was easily performed by pulling the outer portion of the PM tube and suturing the conjunctiva. An uncomplicated XS implantation was performed simultaneously in one patient. For the other patient, a trabeculectomy was performed 1 month after the PM explantation with a good outcome and a functional filtering bleb.

In light of these cases, we would recommend careful screening for and treatment of ocular surface inflammation prior to PM implantation, even if complete control of blepharitis prior to surgery is not always possible in clinical practice. If a deficiency in Tenon’s capsule is noted intraoperatively, scleral fixation of the outer portion of the tube may potentially reduce the risk of further conjunctival perforation. For this purpose, after PM insertion and verification of aqueous humor flow, the external distal part of the tube can be fixed with a 10–0 nylon suture buried within the sclera, preventing the distal end of the tube from pointing towards the conjunctiva. Close monitoring should be performed in such cases because of the higher risk of PM exposure. However, when possible, the distal end of the device should be tucked under a Tenon’s flap, and the Tenon’s layer should be sutured prior to conjunctival closure. As surgical repair is associated with a high rate of recurrence of the PM exposure, as well as a risk of endophthalmitis and bleb fibrosis, we would recommend removal of the device immediately in the case of PM exposure. Another MIGS procedure or filtering surgery can be performed concurrently or as a secondary surgery. However, due to the few cases reported, more studies are required to determine a codified management strategy for PM extrusion.

## Conclusion

PM exposure is a rare surgical issue which requires prompt attention due to the risk of endophthalmitis and complications related to ocular hypotony. Ocular surface inflammation and absence of a Tenon’s flap may play a major role in PM exposure.

## Data Availability

Not applicable.

## Abbreviations

MIGS: Minimally invasive glaucoma surgery
PM: PreserFlo® MicroShunt
SIBS: Polystyrene-block-isobutylene-block- styrene
IOP: Intraocular pressure
XS: Xen Gel stent®
